# Effects of epidural lidocaine analgesia on labor and delivery: A randomized, prospective, controlled trial

**DOI:** 10.1186/1471-2253-6-15

**Published:** 2006-12-18

**Authors:** Shahram Nafisi

**Affiliations:** 1 Department of Anesthesiology and Critical Care, Kashan University of Medical Sciences (KAUMS), Kashan, Iran

## Abstract

**Background:**

Whether epidural analgesia for labor prolongs the active-first and second labor stages and increases the risk of vacuum-assisted delivery is a controversial topic. Our study was conducted to answer the question: does lumbar epidural analgesia with lidocaine affect the progress of labor in our obstetric population?

**Method:**

395 healthy, nulliparous women, at term, presented in spontaneous labor with a singleton vertex presentation. These patients were randomized to receive analgesia either, epidural with bolus doses of 1% lidocaine or intravenous, with meperidine 25 to 50 mg when their cervix was dilated to 4 centimeters. The duration of the active-first and second stages of labor and the neonatal apgar scores were recorded, in each patient. The total number of vacuum-assisted and cesarean deliveries were also measured.

**Results:**

197 women were randomized to the epidural group. 198 women were randomized to the single-dose intravenous meperidine group. There was no statistical difference in rates of vacuum-assisted delivery rate. Cesarean deliveries, as a consequence of fetal bradycardia or dystocia, did not differ significantly between the groups. Differences in the duration of the active-first and the second stages of labor were not statistically significant. The number of newborns with 1-min and 5-min Apgar scores less than 7, did not differ significantly between both analgesia groups.

**Conclusion:**

Epidural analgesia with 1% lidocaine does not prolong the active-first and second stages of labor and does not increase vacuum-assisted or cesarean delivery rate.

## Background

Labor pain is a common phenomenon that can be mitigated by pharmacological and non-pharmacological methods. Epidural techniques can be used to provide complete analgesia during labor [1,2], but some authors believe that epidural analgesia prolongs labor [2-4], and can increase the need for instrumental [1,3,4], or cesarean delivery [5-8].

We performed a controlled trial of 395 nulliparous women who were randomized to either epidural analgesia or single dose intravenous meperidine for pain relief during labor. Our primary purpose was to determine whether the use of 1% lidocaine for epidural analgesia during labor affects duration of second labor stage. Our secondary purposes were the rate of cesarean delivery and neonatal apgar score, vacuum-assisted delivery rate, and duration of active-first stage of labor.

## Methods

The study protocol was developed in collaboration with obstetricians at Kashan University of Medical Sciences (KAUMS) and approved by Kashan University of Medical Sciences ethics commitee. Between June 2004 and February 2005, an independent research assistant approched all nulliparous women that met our study inclusion criteria, when these patients were admitted to the maternity unit in Shabih-Khani Hospital, Kashan, Iran. Eligible patients were provided with written and verbal information about the study. Those patients that did not express interest in a particular form of analgesia were enrolled into the study, after they signed witnessed informed consent.

The inclusion criteria included nulliparity, active labor, cervical dilatation < or = 4 centimeters, single fetus with vertex presentation, ASA Status = 1, and request for analgesia. Exclusion criteria included ASA status ≥2, age <19 years-old, receiving analgesia prior to enrollment, multiparity, probable cephalopelvic disproportion on pelvic examination, and cervical dilatation to >4 centimeters.

Enrolled patients were randomly assigned to receive either epidural or single dose meperidine analgesia during labor. Odd and even numbers were used to allocate patients to either group. All parturients were managed by a nurse anesthetist, under the direct supervision of an anesthesiologist according to the study protocol.

Midwives conducted the obstetric management of all parturients during labor, under the direct supervision of an obstetrician according to the study protocol. Routine intrapartum management of all women included intravenous fluid management and auscultation of the fetal heart with fetal stethoscope or ultrasound fetal heart detector (SONICAID^©^) after each uterine contraction. The frequency and duration of uterine contractions were assessed with the palm of the hand on the uterus. Pelvic examination was performed every hour to evaluate the progress of labor. The aim was to produce a rate of cervical dilation of at least 1 cm/h. When the rate of dilation fell below this, coincidental with hypotonic uterine contractions, oxytocin in a concentration of 10 unit/liter was started at a rate of 6 milliunits/min, increased by 3 milliunits/min at 15-min intervals up to a maximum of 32 milliunits/min and titrated to allow no more than 3 uterine contractions with an acceptable resting duration every 10 minutes.

A Modified Bromage score(1 = complete block, unable to move feet or knee; 2 = almost complete block, able to move feet only; 3 = partial block, just able to move knee; 4 = detectable weakness of hip flexion; 5 = no detectable weakness of hip flexion while supine with full flexion of knees) was obtained before and epidural insertion and again at hourly intervals.

Dystocia was diagnosed when adequate uterine activity did not result in progressive cervical dilation or descent of the fetal head. Indications for the use of vacuum were limited to inadequate voluntary pushing or fetal bradycardia. Inadequate voluntary pushing was determined at the bedside when lack of fetal descent due to inadequate maternal expulsive efforts was observed. In the presence of fetal bradycardia, delivery was expedited when the leading point of the fetal skull was at +2 station.

In the epidural group, once cervical dilatation reached 4 cm, 500 ml of Ringer solution was administered intravenously, and the parturient was seated in the upright position for epidural placement. The low back was prepared and draped in a sterile fashion. An 18-gauge Tuohy needle was inserted at the L3–L4 or L4–L5 interspace with the loss of resistance to air and an epidural catheter (SIMS Portex LTD, UK) was advanced. A 3-ml test dose of 1.5% lidocaine containing epinephrine 15 μg was used to exclude intravascular or subarachnoid placement. A 10-ml bolus of 1% lidocaine was administered via the epidural catheter as a loading dose. Mini-doses of 1% lidocaine were then administered at a rate of 1 ml every 6 min. Additional boluses of 2-ml 1% lidocaine were injected to overcome inadequate analgesia and to achieve a bilateral block between the T-10 and T-8 sensory level. The study ended at the time of vaginal delivery, spontaneous or with vacuum extraction, or when the decision was made to perform a cesarean delivery for any reason. If additional analgesia was needed during labor, 2 ml of 1% lidocaine solution were administered epidurally. If anesthesia was needed for assisted delivery or episiotomy, 5 ml of 2% lidocaine was given. Hypotension, defined as a decrease in systolic arterial blood pressure below 110 mm/Hg, was managed with left uterine displacement, increased intravenous fluid replacement, or 5 mg ephedrine boluses. Following the induction and bolus doses of epidural lidocaine, blood pressures and sensory blockade levels were assessed every minute for 15 minutes and every 10 minutes, thereafter. Maternal oxyhemoglobin saturation and heart rate were monitored with pulse oximetry.

In the single dose meperidine group, meperidine 25–50 mg (~0.5 mg/kg) was given intravenously at 4 cm uterine cervical dilatation.

The mode of delivery (spontaneous, vacuum, cesarean) was recorded. Forceps delivery is not a common procedure in our center.

### Statistical analysis

A power analysis was performed for the primary outcome of interest on the basis of our patients, ie, duration of the second labor stage. On the basis of institutional data, I estimated a sample size of 197 women by group with an assumed standard deviation of 100 minutes, based on my experience significant difference between mean of the groups at least 28 minutes, gives power of 80%, and a 2-tailed alpha error of 0.05. Demographic variables were assessed using descriptive statistics. The Mann-Whitney *U *test was used to compare cervical dilatation rate and second-stage durations. Other outcomes were evaluated using unpaired student *t *test, chi-square analysis, and Fisher exact test as appropriate. *P *< 0.05 was considered significant.

## Results

A total of 395 healthy, nulliparous parturients with a single gestation and an uneventful pregnancy in spontaneous labor were randomized in this investigation. All women completed the study as allocated. None of these patients crossed over to the epidural analgesia group. Women in both groups were similar in both obstetric and demographic characteristics as demonstrated in table 1. Administration of epidural analgesia with 1% lidocaine do not prolong the active-first or second stages of labor significantly, and do not increase the incidence of oxytocin augmentation(Table 2).

**Table 1:** Maternal Demographics Characteristics

**Factor**	**Epidural Analgesia N = 197**	**Meperidine Analgesia N = 198**	**P value**
Age(yr)	23.2 ± 2	22.03 ± 3	NS
Height(cm)	154 ± 9	155 ± 9	NS
Weight(kg)	74 ± 12	74 ± 13	NS
Gestational Age (wk)	38 ± 2	39 ± 1	NS

Data are Mean ± SD Standard Deviation

NS = Not Significant

**Table 2:** Progress of labor

**Labor progress**	**Epidural N = 197**	**Meperidine N = 198**	**P value**
Active phase of first Stage of labor (h)	2.49 ± 1.40	2.40 ± 1.55	NS
Second stage (h)	1.04 ± 0.69	0.86 ± 0.71	NS
Oxytocin augmentation After initiation of Analgesia	197(100)	192(96)	NS
Rate of cervical dilatation (cm/h)	0.53 ± 0.2	0.54 ± 0.2	NS
Fever ≥ 38°C	43(22)	13(7)	<0.001

Data are Mean ± SD Standard Deviation

Or N (%)

NS = Not Significant

There was no difference between groups in the incidence of vacuum-assisted or cesarean deliveries(table 3). The rate of vaginal delivery was not statistically different between the study groups, as well.

**Table 3:** Method of Delivery

**Factor**	**Epidural Analgesia N = 197**	**Meperidine Analgesia N = 198**	**P value**
NVD	169 (85)	175 (88)	NS
VACUUM	4 (2)	4 (2)	NS
C/S (dystocia)	8 (4)	8 (4)	NS
C/S(Bradycardia)	16 (8)	11 (5)	NS

Data are N (%)

NS = Not Significant

As shown in table 4, infant outcome showed no evidence that the type of analgesia had any adverse effects on neonatal Apgar scores. The number of neonates that presented with Apgar scores below 7 at one and five minutes were not statistically different, between both groups. No neonate needed naloxone or ICU admission.

**Table 4:** Apgar Score of Neonates

**Apgar**	**Epidural N = 197**	**Meperidine N = 198**	***P *value**
First minute <7	8	1	NS
Fifth minute <7	7	2	NS

Data are N

NS = Not Significant

We had no crossover from meperidine to epidural analgesia group.

No patient in meperidine group had lower extremity motor weakness at any time as measured by modified Bromage score. All patients from epidural group had a modified Bromage score of 2 throughout labor and delivery.

The preanalgesic visual analog pain scale scores were similar between the two study groups (epidural, 9 ± 1.2 *vs*. intravenous meperidine, 9 ± 1.3; *P *= 0.09). Women who received epidural analgesia reported lower pain scores during the first stage (epidural, 3 ± 3 *vs*. meperidine, 6 ± 4; *P *< 0.0001) and second stage (epidural, 4 ± 3 *vs*. meperidine, 8 ± 2; *P *< 0.0001) of labor. Women who received epidural analgesia had a significantly higher incidence of hypotension compared with women who received intravenous meperidine (30% *vs*. 0; *P *< 0.0001). There was no significant difference in the incidence of nausea and vomiting between the two groups (epidural 6% *vs*. meperidine 4%). There were 5 accidental dural puncture among epidural group (2%). There was no case of incomplete analgesia or urinary retention.

## Discussion

This investigation shows that the well-known muscle relaxant effect of epidural lidocaine did not affect the length of labor in a woman who receives epidural rather than single dose meperidine analgesia. Also, lumbar epidural analgesia using lidocaine was not associated with an increased number of vacuum-assisted or cesarean deliveries in our patient population: an academic hospital setting, in a developing nation.

Since the introduction of inhalational analgesia into obstetric practice in 1847, anesthesia for childbirth has undergone constant change. The popularity of epidural analgesia for childbirth is a recent phenomenon [9]. It is accepted that lumbar epidural analgesia is the most effective method of pain relief in labor, but its putative effects on labor and mode of delivery may influence clinical practice.

Some practitioners will not continue epidural analgesia into the second stage of labor or during childbirth [10], despite lack of data for this practice [11]. They believe that maternal efforts can also be impaired. Conversly, I continue epidural analgesia into the second stage of labor and childbirth, so the mother could be entirely free of pain: pain relief enabled the mother to cooperate in pushing effectively.

Lidocaine is abandoned from epidural labor analgesia because of its motor blocking properties. Epidural analgesia has been said to worsen obstetric outcome because of motor block, and it is thus intuitively clear that lidocaine is not the best drug to use in this situation. However, the evidence linking motor block and obstetric outcome is not as strong as often thought. For example, Evron et al. [12], in a recent prospective, randomized double blinded study showed that the lower intensity of the motor block is not associated with any benefit in terms of obstetric outcomes, duration of the second stage of labor, and obstetric intervention. The only high quality study of the effect of epidural analgesia using lidocaine on obstetric outcomes was by Chestnut *et al*. [13], concluded that maintenance of continuous epidural infusion of lidocaine did not prolong the second stage of labor.

Despite the belief that links motor blocking properties of epidural analgesia to increased instrumental delivery rate, I did not find such correlation. Also there are some studies, in which least concentrated doses of local anesthetic are used for epidural labor analgesia, and patients were able to walk, but instrumentation rate was increased [14].

Bofill *et al*. [15], found that the incidence of instrumental vaginal delivery for dystocia was not increased among epidural patients and that the total increase in instrumental vaginal delivery could have been partially due to the conduct of instrumented deliveries for training purposes. Perhaps there was no correlation between epidural analgesia and vacuum-assisted delivery.

Even though forceps delivery is not a common procedure at our center, our results suggest that the rate of instrumental delivery should not increase with epidural analgesia.

In contrast to my study, Zhang *et al*. [2] found that the rate of vacuum extraction increases notably but the overall rate of instrumental delivery was not increased with epidural analgesia for labor. Conversly, Sharma *et al*. [3] found that significantly more women randomized to epidural analgesia had forceps delivery in comparison with women receiving intravenous opioid. Perhaps such differences are the result of different management and decision making for performing instrumental delivery. The results of the current study suggest that spontaneous delivery is more the result of obstetric management and that epidural analgesia (even with a drug which has not the best theoretical properties) has only minor influence. I continue and sometimes augment analgesia in the second stage of labor and during delivery; therefore a pain free mother can cooperate more fully and can push more effectively. This may work as a, harmless instrumentation, and may neutralize the motor blocking effect of lidocaine. Although this seems true in normal patients, dystotic labor may benefit from the absence of motor block. This however remains to be shown.

Because cesarean delivery carries greater anesthetic and surgical risks than normal vaginal delivery, an increased cesarean section rate secondary to epidural analgesia could adversely affect the opinion of anesthesiologists and obstetricians. Our investigation demonstrated that the rate of cesarean delivery for dystocia or fetal bradycardia is not increased as a result of labor epidural analgesia. Many studies support this conclusion [1-3,16].

A recent systematic review found that epidural analgesia, using low concentraton infusions of bupivacaine, is unlikely to increase the risk of cesarean section [14].

A few reports, prior to 2000 found that epidural analgesia was associated with an increased frequency of cesarean delivery compared with systemic opioid analgesia [5,6]. Most of these reports have methodological flaws or selection biases that contribute to an increased cesarean section rate. Retrospective data collection, to examine the effect of lumbar epidural analgesia on labor outcomes, has a selection bias. For instance, a woman choosing epidural analgesia may be in greater pain; however, this greater pain may be due to a difficult labor, which implicitly poses an increased risk of dystocia and consequently, cesarean delivery [17,18].

In a recent prospective study, Illuzzi et al. [7] found that epidural analgesia was associated with an increased risk of fetal malrotation and cesarean delivery. Prospective studies also have some limitations. Many women have firm views on the type of analgesia they prefer in labor and are reluctant to consent to receive a method of analgesia at random. Women who are well educated, assured, and have mature personalities are at increased risk of obstetric intervention, such as epidural anesthesia [19]. They may choose obstetric care based on perceived access to pain relief [20].

Conversely, some women believe that labor pain is essential to the birthing experience – these patients will forego any analgesic intervention in order to have a completely 'natural' childbirth. They believe that labor pain is God's favor; they would like to acquire spiritual reward, so they are unwilling to receive any analgesic intervention.

The incidence of the cesarean sections in this study is low in both study groups. This may be due to the fact that we initiated analgesia at exact 4 centimeters of uterine cervical dilatation.

Also lidocaine is not as expensive as bupivacaine, the chances of severe complications are less than bupivacaine. Though in cases of delay placement of epidural analgesia, its rapid onset of action compared with bupivacaine is beneficial. Perhaps, for epidural labor analgesia, lidocaine is disproportionately criticized.

There are some limitations to this study: physician assisted delivery of mini-doses of epidural lidocaine may not be practical in developed countries. Nonetheless, in developing countries certain drugs and infusion pumps may not be readily available. A characteristics of this study is the disparity in the quality of pain relief between epidural analgesia and single dose meperidine analgesia groups, which would have been made blinding of clinicians impossible. Additionally, we cannot quantify the impact of a placebo response, engendered by having a physician standing continuously alongside a parturient.

Epidural analgesia for labor is not widely practiced in developing countries and is often not offered to immigrants from the developing world [21-23]. In fact, access to pain relief during labor, in some developing countries, is poor [21]. Patients may demand pain relief, but providers may not recognize the need [21,22]. Even, when providers are willing to provide epidural analgesia, patients in developing countries may resist. In Malaysia, patients refused epidural analgesia because of fear, ignorance, resistance by their husbands, religious reasons, lack of knowledge about the procedure, and poor feedback from friends [24].

In our experience, patients were reluctant to have epidural analgesia because of their desire to have a natural childbirth and their fears about developing back pain and paralysis. Once epidural analgesia was initiated, we used our method of delivery because (1) patients did not want the physician to leave their bedside; (2) epidural pumps were not available; (3) bupivacaine was not available, when the study was initiated; and (4) obstetric co-workers lacked experience with epidurals.

There are some points that make this study stand out from the other trials randomizing laboring women to epidural vs. opioid analgesia: The meperidine dose was quite small. In contrast, most of the randomized trials from the developed world use very large opioid dosage. The cervix was examined hourly. In the developed world, cervical exams are now infrequent. The reported first stage labor lengths therefore are quite accurate. Analgesia was initiated at a uniform cervical dilation. This is unusual in trials from the developed world. While crossovers confound many studies, this study was free from crossover.

Even in those views, which in contrary to mine, insist that epidurals prolong the second stage of labor, it is stated that the length of the second stage, even in those lasting up to 6 hours or more, was not related to infant outcome [25].

In summary, lumbar epidural analgesia with 1% lidocaine does not prolong the active-first and second stages of labor and does not increase the vacuum-assisted or cesarean delivery rate, when compared with intravenous meperidine. Epidural labor analgesia can be delivered, even in communities with significant economic, cultural and religious barriers to this technique. The higher intensity of the motor block of lidocaine was not associated with any harm in terms of obstetric outcome, duration of the second stage of labor, obstetric intervention or neonatal outcome.

## Conclusion

Several studies have helped to confirm the opinion of most anesthesiologists and obstetricians that epidural analgesia only minimally lengthens labor and does not increase the risk of cesarean section.

## Competing interests

The author(s) declare that they have no competing interests.

## Authors' contributions

SN designed and performed the study.

## Pre-publication history

The pre-publication history for this paper can be accessed here:


